# Fully automated plaque characterization in intravascular OCT images using hybrid convolutional and lumen morphology features

**DOI:** 10.1038/s41598-020-59315-6

**Published:** 2020-02-13

**Authors:** Juhwan Lee, David Prabhu, Chaitanya Kolluru, Yazan Gharaibeh, Vladislav N. Zimin, Luis A. P. Dallan, Hiram G. Bezerra, David L. Wilson

**Affiliations:** 1 Department of Biomedical Engineering, Case Western Reserve University, Cleveland, OH 44106 USA; 2 Cardiovascular Imaging Core Laboratory, Harrington Heart and Vascular Institute, University Hospitals Cleveland Medical Center, Cleveland, OH 44106 USA; 3 Department of Radiology, Case Western Reserve University, Cleveland, OH 44106 USA

**Keywords:** Interventional cardiology, Biomedical engineering, Software

## Abstract

For intravascular OCT (IVOCT) images, we developed an automated atherosclerotic plaque characterization method that used a hybrid learning approach, which combined deep-learning convolutional and hand-crafted, lumen morphological features. Processing was done on innate A-line units with labels fibrolipidic (fibrous tissue followed by lipidous tissue), fibrocalcific (fibrous tissue followed by calcification), or other. We trained/tested on an expansive data set (6,556 images), and performed an active learning, relabeling step to improve noisy ground truth labels. Conditional random field was an important post-processing step to reduce classification errors. Sensitivities/specificities were 84.8%/97.8% and 91.4%/95.7% for fibrolipidic and fibrocalcific plaques, respectively. Over lesions, en face classification maps showed automated results that agreed favorably to manually labeled counterparts. Adding lumen morphological features gave statistically significant improvement (p < 0.05), as compared to classification with convolutional features alone. Automated assessments of clinically relevant plaque attributes (arc angle and length), compared favorably to those from manual labels. Our hybrid approach gave statistically improved results as compared to previous A-line classification methods using deep learning or hand-crafted features alone. This plaque characterization approach is fully automated, robust, and promising for live-time treatment planning and research applications.

## Introduction

Intravascular optical coherence tomography (IVOCT) is an important technology for planning and assessment of interventional, percutaneous treatments of coronary artery disease. IVOCT is a high contrast, high-resolution imaging modality that uses near-infrared light^1^. Compared to intravascular ultrasound (IVUS), this modality provides better image resolution with axial resolution ranging from 12 to 18 *μm* (as compared to 150–250 *μm* from IVUS) and lateral resolution ranging from 20 to 90 *μm* (as compared to 150–300 *μm* from IVUS)^1^. IVOCT allows to determine different plaque components such as fibrous, lipidous, and calcified tissues, and is the only modality that can identify thin cap fibroatheroma^2^. IVOCT is used for clinical, live-time intervention planning, and stent deployment assessment. IVOCT-guided percutaneous coronary intervention (PCI) brings valuable benefit for patient treatment as compared to PCI guided by X-ray angiography alone^3^. In addition, IVOCT is used for clinical research studies such as the calcium scoring analysis^4^ and calcium crack formation^5^.

Although IVOCT is clearly an excellent method for intravascular imaging of plaque, it has limitations. One is the cost of transducers. Another is tissue penetration depth, especially in the presence of lipidous plaque. Another is the need for a physician trained in visual interpretation of IVOCT images who is willing to take the time to examine images during a stressful procedure. A single IVOCT pullback typically generates 300–500 image frames resulting in data overload. Even when IVOCT is used for research purposes, manual labeling of plaque is labor-intensive, requiring up to 1 day for each pullback by a trained cardiologist. Manual labeling is also prone to inter- and intra-observer variability. In prior work^6^, our group found evidence of up to 5% intra- and 6% inter-observer variability among cardiologists detecting stent struts in IVOCT images. To address the limitation of visual interpretation, researchers are creating automated software.

There are reports of fully automated plaque characterization approaches based on machine and deep learning methods. Ughi *et al*.^7^ suggested a fully-automated plaque characterization method using the statistical features and random forest classifier in IVOCT images. They applied the pre-processing step to remove the guidewire and to segment the lumen in (*r, θ*) IVOCT images. Classification accuracies were 89.5%, 79.5%, and 72.1% for fibrotic, lipid-rich, and calcified tissues, respectively. Athanasiou *et al*.^8^ automatically detected four plaque types using a set of intensity and texture-based features with the random forest classifier. Classification sensitivities were 81%, 71%, 87%, and 81% for calcium, lipid tissue, fibrous tissue, and mixed tissue, respectively. Rico-Jimenez *et al*.^9^ extracted the 11 morphological features including the A-line peaks and signal decay, and automatically classified each A-line as one of either intimal thickening, fibrotic, superficial lipid, and fibrotic-lipid based on a linear discriminant analysis algorithm. Some groups have reported that the optical attenuation coefficient^7,10–12^ of the tissue is useful for characterizing coronary plaques. Recently, some researchers have attempted to implement deep learning techniques for plaque characterization. Yong *et al*.^13^ proposed a linear regression convolutional neural network (CNN) model to automatically segment the vessel lumen. Their network consisted of four convolutional layers and three fully connected layers, including the output layer. They evaluated their method on 64 pullbacks acquired from 28 patients and achieved mean locational accuracy of 22 microns with very fast computation time (40.6 ms per image). To automatically determine vessel borders and coronary plaques (e.g., fibrosis and calcification), Abdolmanafi *et al*.^14,15^ used the pre-trained CNN models (e.g., AlexNet^16^, VGG-19^17^, and Inception-v3^18^) for extracting deep learning features and combined those features to train the machine learning model. He *et al*.^19^ extracted the plaque tissue area and employed a simple CNN model to automatically classify each pixel into one of the five categories: lipid tissue, fibrous tissue, mixed tissue, calcified tissue, and background. Athanasiou *et al*.^20^ classified the whole arterial wall into six tissue types: calcium, lipid tissue, fibrous tissue, mixed tissue, non-pathological tissue, and no visible tissue using a CNN model having 45 layers. An overall accuracy was 96.1%. More recently, Gessert *et al*.^21^ used two state-of-the-art deep learning models, ResNet50-V2^22^ and DenseNet121^23^ for automated plaque detection. The ResNet was used for a better gradient flow and improved optimization, and the DenseNet was focused on efficiency of classification. Overall accuracy and sensitivity were 91.7% and 90.9% with high F1 score (0.913). Zhang *et al*.^24^ proposed an automated plaque segmentation method based on the simple CNN and an improved random walk algorithm. They obtained the same Jaccard coefficient of 0.864 for lipid and calcified plaque segmentation. Abdolmanafi *et al*.^25^ used the VGG-19 model^17^ as the encoder of the fully convolutional network to identify the calcification, fibrosis, macrophage, and neovascularization. An overall sensitivity of 89.3% was reported. Most recently, our group developed a fully automated plaque characterization method for lipidous and calcified plaques based on semantic segmentation using the SegNet deep learning model^26^. We obtained the sensitivities/specificities of 87.4%/89.5% and 85.1%/94.2% for lipidous and calcified plaques, respectively. We also provided A-line classification maps based on which plaque was in the majority in each A-line. In a previous report, we obtained good results using A-line classification with a CNN^27^. In another report, we used a conventional machine learning approach and evaluated over 1,000 features, including a set of lumen morphology features, for classification of A-lines^28^. This variety of publications suggests that plaque classification is an important problem and that there are a variety of different approaches that should be evaluated. Some of these details are summarized in Table S1.

In this paper, we build on our previous studies and develop an automated, A-line-based plaque characterization method by combining A-line deep learning and hand-crafted, lumen morphology features. Such hybrid learning approaches, which combine deep learning and hand crafted features, have been successfully applied to multiple problems^29–32^. Each A-line will be classified as fibrolipidic (fibrous tissue at the lumen border followed by lipidous tissue), fibrocalcific (fibrous tissue followed by calcification), or other. We perform image processing procedures in the polar (*r, θ*) domain to avoid interpolation effects seen in the Cartesian (*x, y*) domain. In addition to conventional quantitative classification metrics, we measure clinically meaningful plaque attributes (arc angle and length). We limit our classification targets to lipidous (fibrolipidic) and calcified (fibrocalcific) plaques, since these types of plaques influence clinical decision making^33–35^.

This study has several important aspects and contributions. First, A-line classification avoids the issue of the indeterminate back border of lipidous plaque found in pixel classification methods. That is, because there is rapid absorption of light in lipidous tissues, one cannot determine the location of the back boarder. Second, we create a powerful, hybrid classification which combines deep learning A-line features and hand-crafted lumen morphological features. The latter exploits the observation that non-circular lumens are often observed in the presence of calcifications^28^. Essentially, we are combining two methods previously reported by us: A-line CNN classification^27^ and lumen morphological features which were identified as important among 1000 s of hand-crafted features previously reported^28^. Third, we use a large number of IVOCT images and corresponding labels, including a wide variety of lesions (i.e., over 6,500 images, 49 patients, and 111 volumes of interest (VOIs)). Having a large variety of lesions is important to ensure generalization. Fourth, in addition to conventional segmentation metrics (e.g., F1 score), we measure clinically meaningful plaque attributes (arc angle and length) for performance evaluation. Fifth, we apply an active learning experiment to possibly reduce inconsistent labeling, which could arise due to analyst fatigue or criteria drift. In this, a highly trained, independent analyst is presented with a number of images containing discrepancies and asked to label them again, blind to previous labels and computer predictions.

## Image Analysis Methods

Our A-line plaque classification method involved several steps. First, we preprocessed (*r, θ*) data to identify the tissue region of interest and to reduce noise. Second, deep learning CNN features, optimized for A-line classification, were extracted for each A-line. Third, lumen morphological features were extracted from both (*r, θ*) and (*x, y*) data. Fourth, a random forest classifier, which used a hybrid of deep learning and hand-crafted features, was used to classify A-lines as fibrocalcific, fibrolipidic, or other. Fifth, CRF was used to reduce classification errors. Learning was done using standard approaches.

### Pre-processing

Pre-processing of (*r, θ*) image data consisted of several steps, principally to identify the appropriate tissue region of interest for processing. First, to remove the guidewire and its shadow regions, we created an accumulated intensity map by adding all the pixel values along *r* in each A-line, as the guidewire usually generates a long dark shadow along the radial direction. Then, we detected the shadow regions using dynamic programming^36^ and removed it. Second, we identified the lumen boundary using dynamic programming^36^. Briefly, we computed the image gradient and regarded the contour with the highest cumulative edge strength as the lumen boundary. Third, each A-line was pixel-shifted to the left (smaller *r*) allowing all A-lines to have the same starting pixel. Fourth, we designated the first 200 pixels (~1 mm) in the *r* direction as the region of interest for processing, due to the limited penetration depth of IVOCT. Finally, we applied a Gaussian filter to reduce noise, using a kernel with standard deviation 1 and footprint of (7, 7).

### Feature extraction

#### Extraction of convolutional features

**Figure 1:**
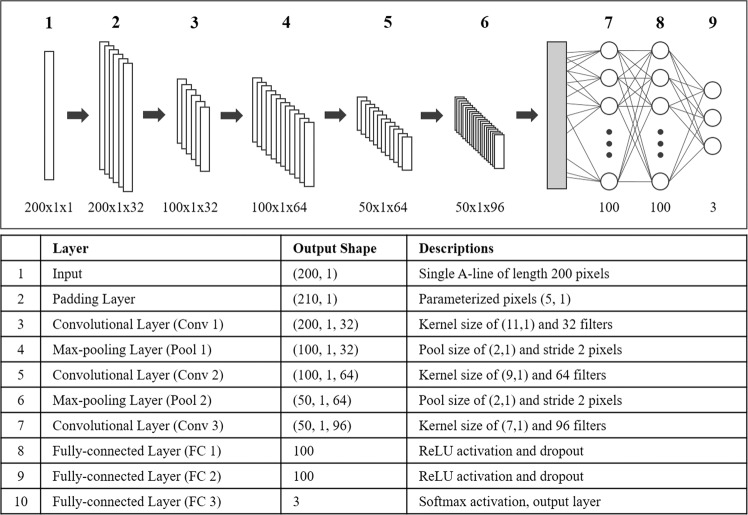
CNN architecture for convolutional feature extraction. The network consists of three convolutional (Conv 1–3), two max-pooling (Pool 1–2), and three fully-connected layers (FC 1–3). Before the Conv-1 layer, we added the padding layer which adds pixel values at the left and right edges, along the *r* direction. Processing was done in the (*r, θ*) view. Details of each layer are provided in the table and Section 2.2.

We created a CNN to classify A-lines, trained it, and then used the network to compute features from IVOCT images for machine learning, random forest processing. The CNN was trained to classify A-lines as fibrocalcific, fibrolipidic, or other. The CNN model consisted of three convolutional, two maximum pooling, and three fully connected layers (Fig. 1). Each convolutional layer consisted of convolutional, batch normalization, and rectified linear unit (ReLU) layers. Before processing, we padded the left and right edges of the input A-line by 5 pixels (Layer 1). The left padding was set to zero because those regions belong to the luminal area (an anatomical feature of interest), while the five pixels after region of interest (201–205 pixels in r axis) were used for the right padding. In our model, convolutional layers had a varying number of filters (32, 64, and 96) and kernel size (11, 9, and 7) and generated the relevant feature maps with a stride of 2 pixels. After convolutional operation, batch normalization and ReLU layers were added to accelerate training process and reduce the sensitivity to network initialization^16^. The maximum pooling layer with a pool size of 2 pixels was subsequently implemented for dimensionality reduction. This layer enabled to prevent overfitting, and kept the network invariant to small transformations, distortions, and translations^16^. The three fully connected layers were followed by pairs of convolutional and pooling layers. The first two layers included 100 output units with ReLU activation functions and dropout layers, while the Softmax activation was used along with three output units for the last layer. After training, we removed the last fully connected layer and obtained 100 convolutional features from the activations of the previous fully connected layer. These features were later combined with lumen morphology features.

#### Lumen morphology features

**Figure 2:**
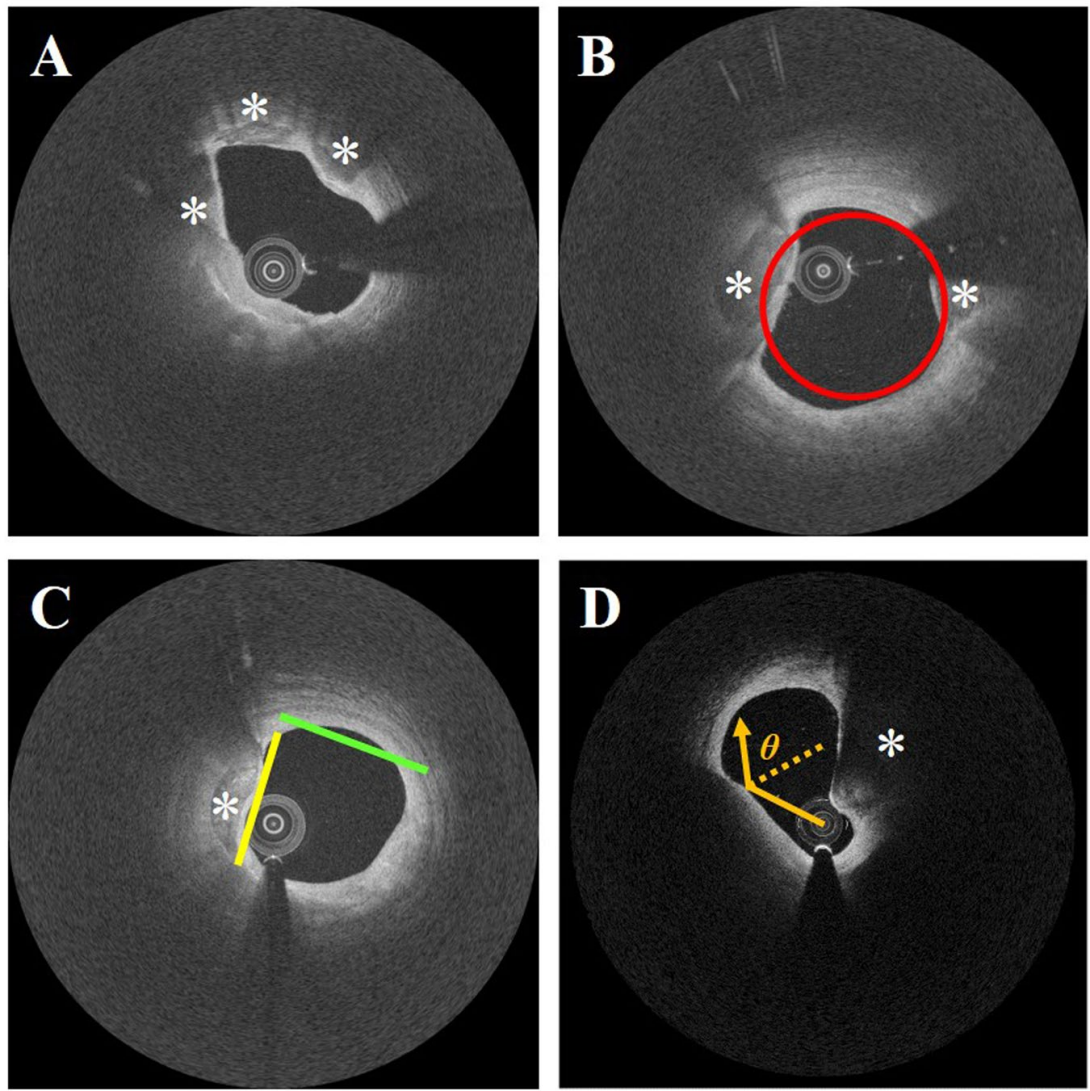
Lumen morphology features. Panels show: (**A**) very eccentric lumen with 3 calcified lesions, (**B**) superimposed circular lumen boundary (red) having the same area as the lumen, centered at the center of mass, (**C**) best-fit line (yellow and green) using 1/8 of the points around the lumen border, and (**D**) superficial attenuation (orange) of IVOCT signal caused from oblique beam incidence due to an eccentric catheter. White asterisk represents the calcified lesions. Here and in other figures, IVOCT images are shown following log transformation for improved visualization.

We extracted lumen morphological features using methods described previously^28^, which are now only briefly described. We estimated the change in lumen area by analyzing the lumen area of current frame to each of its adjacent ± 3 frames. As shown in Fig. 2A, we computed the lumen eccentricity of current frame as well as the change in lumen eccentricity across adjacent ± 3 frames. Individual A-line lumen eccentricity was also obtained to localize A-lines containing plaque (Fig. 2B). To determine this, we created a concentric circle having the same center of mass and area as the segmented lumen. Then, we measured the signed distance between the boundary pixel of each A-line and the corresponding circle boundary pixel along the radius from the center of mass. In addition, we found that large calcifications often tend to cause a flat lumen in our dataset, especially when they are close to the vessel wall. Therefore, we generated a best-fit line for lumen boundary (Fig. 2C) and extracted relevant features: sum of squared residuals, goodness of fit, and line magnitude. For the previously described lumen features, we also observed the change in these features across adjacent ± 3 frames. Furthermore, because of the physics of light reflection, a signal-poor region with diffuse outer border can appear, when the IVOCT signal is obliquely incident on the tissue surface^37^. To help identify such regions, we computed the R-θ lumen curvature (Fig. 2D) by assessing the lumen slope, where a slope of 0 indicates perpendicular incidence of the beam, and a large positive or negative value indicates an oblique intersection. In total, we extracted 371 morphological features in IVOCT images. The details of these features are referred in^28^.

### Random forest classification

We used the random forest classifier as it has several advantages for the analysis of IVOCT images. First, this method works efficiently on a large number of input attributes (features) with a low risk of overfitting. Second, it is fairly robust for noisy data. Third, it can provide the relative importance of features in determining classification, thus requiring no separate feature selection step. Fourth, learning and computation time is fast, even for very large data sets. The numbers of trees and variables are the two most important parameters for determining classification performance of random forest classifier^38^. The optimum numbers of classification trees and variables were determined by considering the out-of-bag error (OOB) data which are not used in bootstrap sampling. Implementation details are described in Section 3.2.

### Classification noise reduction using fully connected conditional random field (CRF)

Since A-line classification does not fully consider spatial similarities between nearby A-lines in *θ* and *z*, we employed classification noise cleaning. In particular, we implemented a fully connected CRF that defines the pairwise edge potentials of each probabilistic classification (*θ, z*) by a linear combination of Gaussian kernels^39^. CRF is not only able to model arbitrarily large pixel-neighborhoods, but also computationally efficient, making it ideal for IVOCT plaque characterization. This method plays a very important role in performance improvement as it enables us to consider the spatial similarities.

In a fully connected CRF, there are two Gaussian kernel potentials, which are smoothness and appearance kernels:

1 $${\rm{k}}({f}_{i},{f}_{j})={\omega }_{1}exp(-\frac{{|{p}_{i}-{p}_{j}|}^{2}}{2{\theta }_{\alpha }^{2}})+{\omega }_{2}exp(-\frac{{|{p}_{i}-{p}_{j}|}^{2}}{2{\theta }_{\beta }^{2}}-\frac{{|{I}_{i}-{I}_{j}|}^{2}}{2{\theta }_{\gamma }^{2}})$$

where *p* and *I* are the spatial position and the intensity vectors, respectively. The configurable parameter *θ*_*α*_ expresses the size and shape of neighborhoods. The parameters *θ*_*β*_ and *θ*_γ_ control the degrees of nearness and similarity. The configurable weights *ω*_1_ and *ω*_2_ define the relative strength of the two factors. The smoothness kernel (first term) can be expressed by a diagonal covariance matrix and removes small isolated regions in the predicted results^40^. The appearance kernel (second term) performs optimization based on the observation that adjacent pixels with similar intensity values are likely to be in the same class. Since pixels in the en face image have no specific intensity value, the appearance kernel was dropped by setting ω_2_ to zero. Therefore, our implementation of CRF only included the smoothness kernel. Detailed descriptions and equations are referred in^39^.

## Experimental Methods

### Images and their annotation

IVOCT images were acquired with a frequency-domain OCT system (ILUMIEN OPTIS; St. Jude Medical Inc.). During image acquisition, the optical probe was automatically pulled back from distal to proximal at a speed of 36 *mm/s*, frame rate of 180 *frames/sec*, and axial resolution of 20 *μm*. All IVOCT image frames were initially reviewed, and frames having poor quality due to luminal blood, unclear lumen, artifact, or reverberation were excluded. We used a total of 6,556 image frames across 49 patients with 111 VOIs having calcification (34 VOIs), lipidous regions (37 VOIs), or both (13 VOIs), and 27 VOIs without any identified calcification or lipidous region. The sizes of raw (*r,θ*) data before processing were either 968 × 496 or 968 × 448 pixels in 16-bit gray scale. We used every IVOCT pullback that was considered analyzable by cardiologists. The images with stents and poor quality were excluded to avoid misleading results.

**Figure 3:**
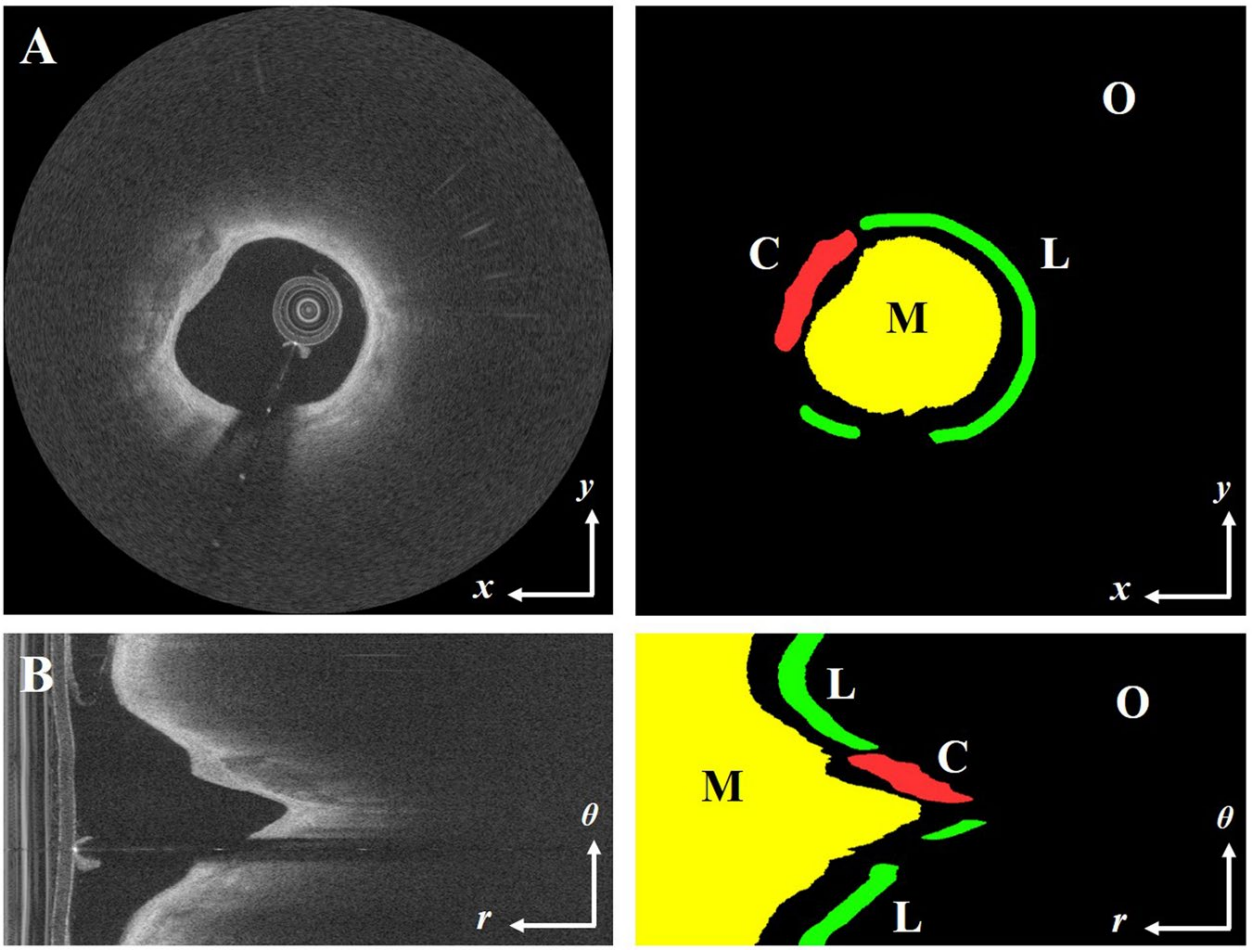
Example annotations of IVOCT images in (**A**) (*x, y*) and (**B**) (*r, θ*) view. The left and right figures indicate IVOCT image and corresponding manual annotation, respectively, with labels (M: lumen, L: lipid, C: calcium, and O: other). As described in Section 3.1, the A-line labels of fibrolipidic, fibrocalcific, and other classes were created by counting the number of pixels in each A-line.

Each A-line from each VOI was manually annotated as now described. Log compressed, Cartesian (*x,y*) images were analyzed by two experienced readers from Cardiovascular Imaging Core Laboratory of Harrington Heart and Vascular Institute (University Hospitals Cleveland Medical Center, Cleveland, OH, USA), a laboratory that has done numerous studies requiring expert reading of IVOCT images. Using definitions in the “consensus document”^2^, they manually labeled regions containing lipidous and calcified plaques. Tissue pixels without annotation were considered others. In case of disagreement, readers revaluated the frames and reached a consensus decision. From a simple rule, we next created A-line labels, consisting of fibrolipidic, fibrocalcific, and other, as reported previously^27,28^. If an A-line included ≥3 pixels of either lipidous or calcified plaques, we determined which of these two classes was in the majority, and then labeled the A-line accordingly as fibrolipidic or fibrocalcific. All other A-lines were identified as being in the “other” class. Figure 3 shows an example annotation containing lipidous (fibrolipidic), calcified (fibrocalcific), and other classes. Examples of lipidous and calcified plaques are shown in IVOCT images and corresponding cryo-images in Fig. S1 in the Supplementary Material.

### Implementation and optimization of the CNN and random forest

The CNN and random forest were trained on the exact same VOIs from the five-fold cross validation. For the CNN classifier, we minimized a cross-entropy loss function over the softmax outputs. Although the CNN model was not used for final classification, overfitting could still degrade the reliability of convolutional features. To limit overfitting, we set the training to stop when the validation loss did not improve over 5 consecutive epochs, or when the number of epochs reached a pre-defined value (50). The former stopping criterion was typically exercised. Because data were imbalanced, we modified the loss function to include relative class weights of each class as determined from the inversed median frequency of class proportions. The CNN model was trained using adaptive moment estimation (ADAM) optimizer^41^. ADAM optimization is an extension to stochastic gradient descent optimizer that only requires first-order gradients with little memory requirement. This method computes individual adaptive learning rates for different parameters and is well-suited to a non-convex optimization problems^41^. All parameters of the CNN model were initialized with random Gaussian distributions. The model was trained with a batch size of 30 for 50 epochs, momentum with decay of 0.9, base learning rate of 0.001, learning rate drop factor of 0.2, and learning rate drop period of 5. The size of the receptive field of CNN model was 77 × 1 pixels. The training progress curve on the one-fold cross validation data set is shown in Fig. S2 in the Supplementary Material. The random forest classifier was optimized. We varied the number of trees from 100 to 1,000 in intervals of 50 and compared OOB error rates. The error rate decreased to a nearly constant value at about 250 trees (Fig. S3), and we set the number of trees to 250 in all other analyses. Similarly, the number of variables was empirically identified to be 16. Image processing and network training were performed using MATLAB software package (R2018b, MathWorks Inc.) on a NVIDIA GeForce TITAN RTX GPU with 120GB of RAM installed in a Dell Precision T7610.

### Performance evaluation

To evaluate classification performance and its variation across samples, we used five-fold cross validation. Among the entire data sets, 89 VOIs were divided into five independent subsets, consisting of 15–22 VOIs each, and during each of the 5 folded evaluations, 3 were assigned for training, 1 for validation, and 1 for testing. This process was repeated five times such that all pullbacks were used in a test set. We determined subsets based on VOIs rather than images, as the latter can leave very similar images in training and testing sets, giving unrealistic performance. The rest (22 VOIs) were used as held-out data for further evaluations. These images were neither used for training nor testing during cross validation. When we compared processing methods, the exact same folds were utilized. Classification performance was quantitatively evaluated using traditional classification metrics as given below:

2 $$Sensitivity=\frac{TP}{TP+FN}$$

3 $$Specificity=\frac{TN}{TN+FP}$$

4 $$F1\,Score=\frac{2TP}{2TP+FP+FN}$$

**Figure 4:**
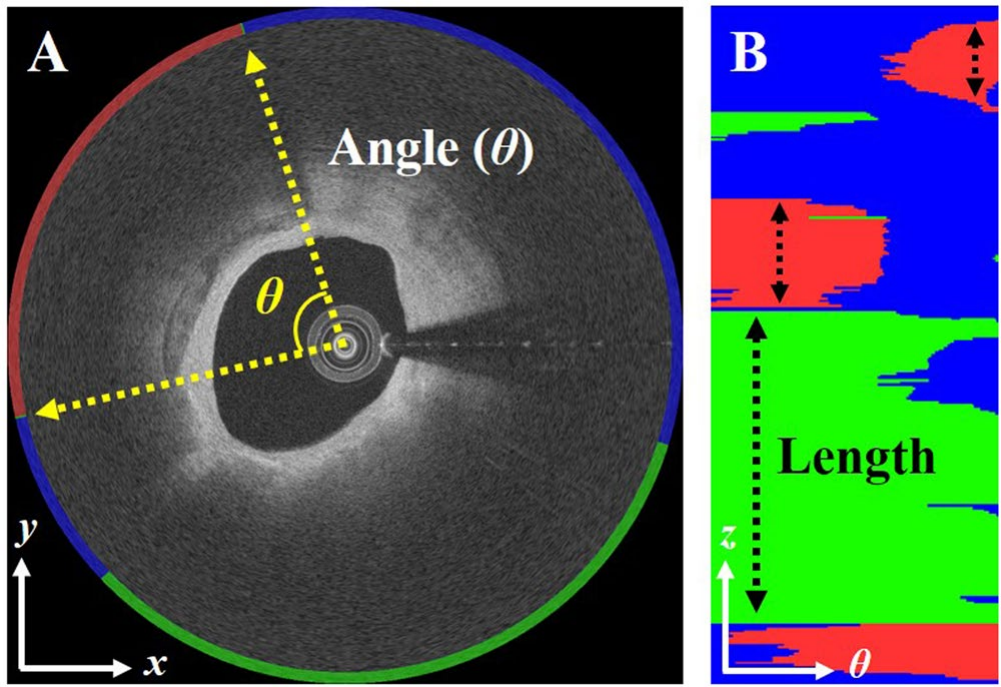
Clinical plaque attributes: (**A**) angle and (**B**) length. Angle and length were measured in (*x, y*) and *en-face* (*θ, z*) images, respectively. Specifically, we computed the plaque length in each *θ*-line and compared results between the manual and proposed methods. Colors are green (fibrolipidic), red (fibrocalcific), and blue (other).

Above, *TP* is the number of true positives, *TN* is the number of true negatives, *FP* is the number of false positives, and *FN* is the number of false negatives. In addition, we also measured clinical plaque attributes, such as arc angle and length, to provide a more clinically meaningful analysis (Fig. 4). The arc angle was measured from the center of the catheter to the boundaries of plaque A-lines. The length was the total number of frames containing a contiguous plaque multiplied by the frame interval. These attributes were evaluated using Bland-Altman analysis.

### Active learning relabeling

The ground truth labels of IVOCT images used in this study were manually created by two expert readers. Although they are consistently trained and had similar experience, there could be inconsistent criteria applied over time. Therefore, we performed an active learning experiment as follows. Following training on all data, we selected the 2 folds having a low mean F1 score (<0.7) and asked an experienced cardiologist to relabel them. During relabeling, the experienced cardiologist was completely blinded to previous manual and automatic labels. After relabeling, we performed the five-fold cross validation again to determine any improvements.

## Results

**Figure 5:**
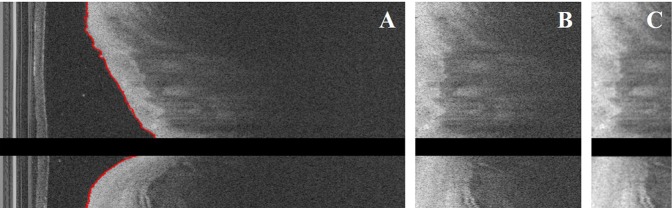
Pre-processing result for a representative IVOCT image. Panels show: (**A**) lumen segmentation (red) using dynamic programming and guidewire removal, (**B**) pixel-shifted image, and (**C**) determination of ROI (~1 *mm*) and speckle noise reduction using Gaussian filtering. (**B**,**C**) were cropped. After pre-processing, the size of the IVOCT image was changed to 200 × 496 pixels, and the convolutional and lumen morphology features were extracted from these data.

There was processing prior to classification. Tissue regions were identified for further processing (Fig. 5). The lumen boundary (red) was accurately segmented, and guidewire and corresponding shadow regions were removed (Fig. 5A). The result was pixel shifted to the left (Fig. 5B). This normalized lesion appearance across training data. The region of interest was determined and filtered to reduce speckle noise (Fig. 5C). The latter image was used as the input to the CNN. The CNN was trained to classify A-lines as part of the train/test/validate process across the five folds. Once trained, the CNN was stripped down to create features for further hybrid processing.

**Figure 6:**
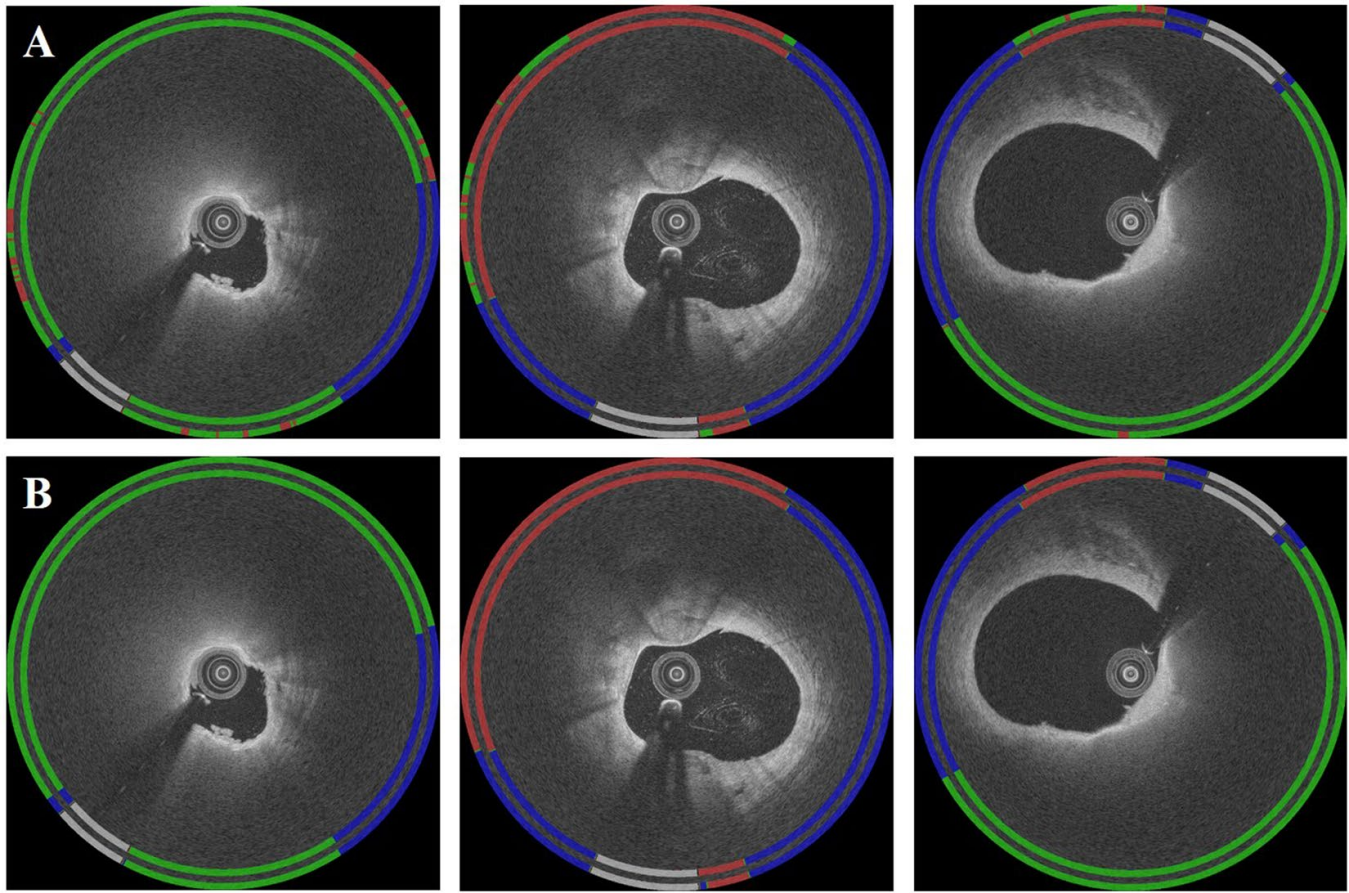
A-line classification results before and after CRF noise cleaning mapped to (x, y) domain. Panels are: (**A**) results prior to noise cleaning and (**B**) results after noise cleaning. For each figure, the inner ring is the ground truth label, and the outer ring is the predicted result. Colors are green (fibrolipidic), red (fibrocalcific), blue (other), and white (guidewire).

Hybrid classification followed by CRF gave good A-line classification results. Figure 6 demonstrates a representative classification result from one of the folds. Collecting results across the five folds, most traditional metrics (i.e., sensitivity and F1 score) were significantly improved for all classes after classification noise cleaning (p < 0.05) (Table 1). The proposed method gave better classification results for the fibrolipidic class (F1 score ≈ 0.887) as compared to those in fibrocalcific class (F1 score ≈ 0.677).

**Table 1:** Mean metrics over the five folds before, and after CRF noise cleaning, and with active learning.

Classes		Sensitivity (%)	Specificity (%)	F1 score
Fibrolipidic	Before noise cleaning	66.0 ± 20.9	93.5 ± 5.9	0.727 ± 0.174
	After noise cleaning	84.2 ± 9.5*	97.7 ± 1.7	0.882 ± 0.049*
	After active learning	84.8 ± 8.2*	97.8 ± 1.6	0.887 ± 0.040*
Fibrocalcific	Before noise cleaning	61.1 ± 27.0	94.0 ± 2.0	0.530 ± 0.184
	After noise cleaning	91.4 ± 6.9*	95.7 ± 1.8	0.677 ± 0.119*
	After active learning	91.2 ± 6.4*	96.2 ± 1.6	0.719 ± 0.070*†
Other	Before noise cleaning	95.0 ± 7.3	80.9 ± 26.5	0.921 ± 0.108
	After noise cleaning	96.7 ± 1.6	98.1 ± 0.8*	0.979 ± 0.010*
	After active learning	96.5 ± 1.1	97.4 ± 0.6*	0.977 ± 0.008*

Sensitivity and F1 score of fibrolipidic and fibrocalcific plaques were significantly improved after CRF noise cleaning. Statistically significant differences (p < 0.05) compared with ‘before CRF’ are indicated by an asterisk (*), as determined from the Wilcoxon signed-rank test. After active learning, the F1 score improved from 0.677 to 0.719, and significantly changed (†p < 0.05).

Active learning improved results. To reduce classification errors, a most experienced cardiologist very carefully reexamined the calcified plaque lesions of two folds (33 VOIs, 1,332 images), with low mean F1 scores (<0.7). Please note that relabeling was done in a very conservative manner. The experienced cardiologist was naïve to automated segmentation results and did not see automated or initial manual annotations during this labeling step. In the 33 VOIs and 1,332 images, most changes between initial and secondary labels were at the edges of a lesion. The experienced cardiologist added calcifications to 16 images not previously identified as having calcification. The mean F1 score of fibrocalcific plaque was improved from 0.677 to 0.719 (Table 1), a changed determined to be significant (p < 0.05). There was only a small, insignificant difference in the fibrolipidic and other classes. Because we now had initial and secondary labeling on two folds, this enabled additional analyses. Assuming the secondary labeling to be the gold standard, we determined an F1 score for fibrocalcific of 0.965 and 0.937 for the two folds. Comparing the automated test results from these two folds, we found similar scores (i.e., 0.912 and 0.981, respectively).

**Figure 7:**
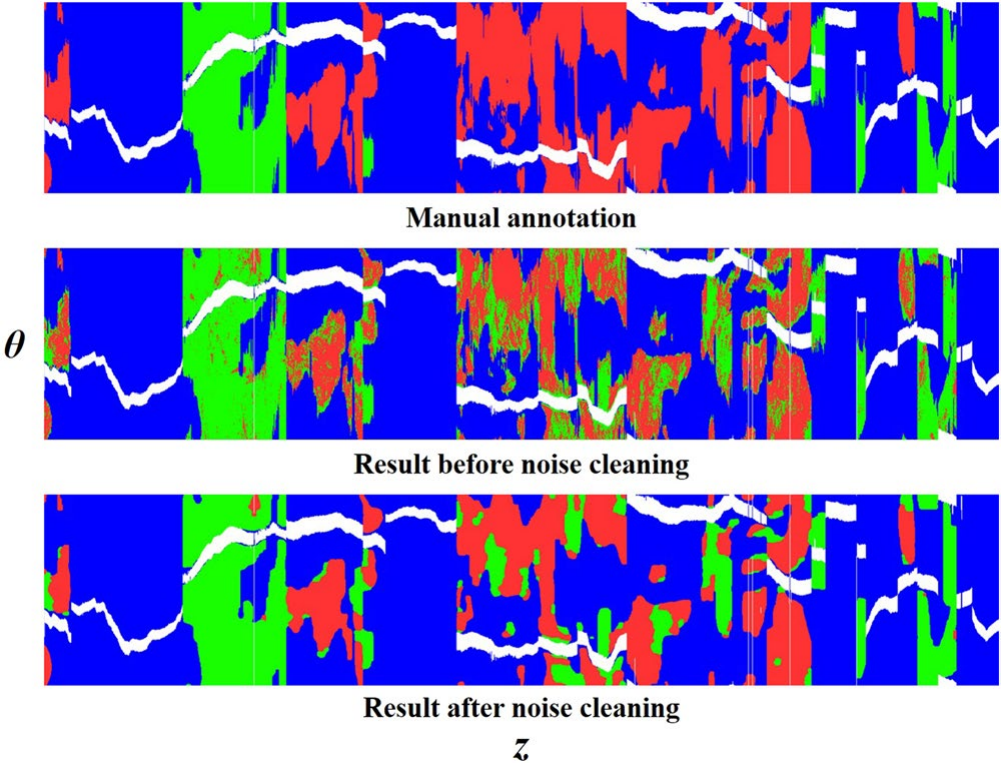
A-line classification results in en face (*θ, z*) view. Panels are: (top) manual annotation, (middle) results prior to noise cleaning, and (bottom) result after noise cleaning. Colors are green (fibrolipidic), red (fibrocalcific), blue (other), and white (guidewire). Results were created by concatenating results across all 22 VOI test results from the held-out set.

To further evaluate processing, we trained our hybrid method using the entire 89 VOIs (4,819 images) and tested on the held-out data (22 VOIs, 1,737 images). Performance was similar to that in the fold analysis (Tables 1 and 2), i.e., nearly within the standard deviation of the folds’ analysis. Fibrocalcific plaque showed higher sensitivity compared to fibrolipidic plaque, while the F1 score and specificity were higher in fibrolipidic class. Since plaques are spatially distributed in coronary artery, we created the en face (*θ, z*) map of the predicted A-line results (Fig. 7). The predicted classification maps agreed favorably to the manually annotated counterparts. The confusion matrix is shown in Fig. S4 in the Supplementary Material.

**Table 2:** Effect of adding hand-crafted morphological features to CNN features.

Classes		Sensitivity (%)	Specificity (%)	F1 score
Convolutional Features	Fibrolipidic	46.8	97.7	0.610
	Fibrocalcific	80.2	92.9	0.800
	Other	93.5	67.1	0.847
Lumen Morphological Features	Fibrolipidic	63.0	94.8	0.674
	Fibrocalcific	100	76.3	0.011
	Other	75.2	97.8	0.857
Hybrid Features	Fibrolipidic	77.3*†	98.9	0.845*†
	Fibrocalcific	97.2*	91.9†	0.816*†
	Other	93.7†	97.4*	0.962*†

We report metrics for the held-out test set obtained using only CNN features (top), only lumen morphology features (middle), and hybrid features (bottom) after post processing. For fibrolipidic and fibrocalcific classes, sensitivity and F1 score were significantly improved with hybrid features as compared to CNN features (*p < 0.05) and lumen morphology features (†p < 0.05). The Wilcoxon signed-rank test was performed. In this experiment, we used all images available to training without a folds’ analysis, as was done in Table 1. Exactly thesame training (89 VOIs, 4,819 images) / testing (22 VOIs, 1,737 images) images were used for this comparison.

**Figure 8:**
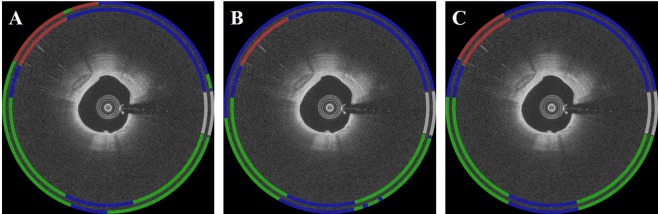
Comparison of classification results obtained using (**A**) only convolutional, (**B**) only lumen morphology, and (**C**) hybrid features. The inner ring is the ground truth label, and the outer ring is the predicted result. Colors are green (fibrolipidic), red (fibrocalcific), blue (other), and white (guidewire).

On the held-out data set, we determined the effect of adding hand-crafted morphological features by comparing the CNN classifier to the hybrid classifier. In Fig. 8, the convolutional features showed numerous false negatives, especially for fibrocalcific plaque as compared to the ground truth. Lumen morphology features were hardly able to detect fibrocalcific plaque (F1 score = 0.011). In Table 2, the hybrid classifier significantly outperformed the CNN classifier, indicating the importance of hand-crafted morphological features. Improvements were obtained both for fibrocalcifican and fibrolipic A-lines.

**Figure 9:**
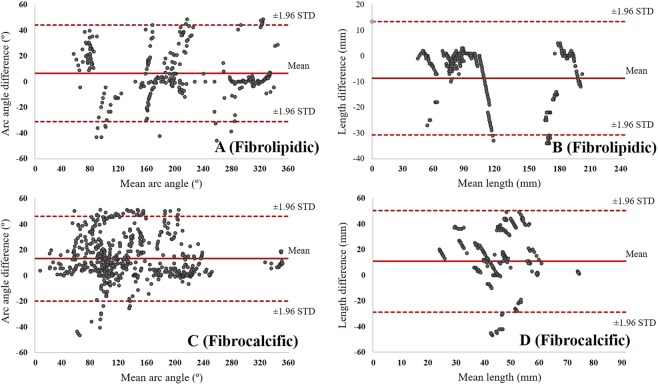
Bland-Altman plot demonstrates strong correlations of clinical plaque attributes (arc angle and length) between the manual and automated results. (**A**,**B**) show arc angle and length of fibrolipidic plaque. In both instances, the vertical axis is (predicted – actual). (**C**,**D**) show similar analyses of fibrocalcific plaque. Mean values of (**A**–**D**) were 6.5 ± 19.2°, −8.7 ± 11.3 mm, 13.2 ± 16.8°, and 10.8 ± 20.1 mm, respectively. The arc angle and length were measured as shown in Fig. 4.

Bland-Altman analysis was performed to evaluate the statistical agreement of measured clinical plaque attributes (i.e., arc angle and length) between manual and automated methods. In Fig. 9, the fibrolipidic plaque had much lower biases of 6.5 ± 19.2° (angle) and −8.7 ± 11.3 mm (length) as compared to the fibrocalcific plaque having 13.2 ± 16.8° (angle) and 10.8 ± 20.1 mm (length). Although the fibrocalcific class had a relatively large bias, most of points were included in the limit of agreement. These promising results indicate that the proposed method is well-suited for plaque characterization in IVOCT images.

The proposed hybrid method was compared to our two previous A-line-based classification approaches which used deep learning^27^ and hand crafted features^28^ alone. We trained/tested the previous approaches on the exact same data sets (five folds for training and held-out for testing) used in this study. As shown in Table 3, the proposed method was statistically different and better than the previous methods for all plaque types.

**Table 3:** Comparison of current method to the previously reported CNN-based (CK)^27^ and morphological feature-based (DSP)^28^ approaches.

Classes		Sensitivity (%)	Specificity (%)	F1 score
Fibrolipidic	CK Method	52.2	97.9	0.656
	DSP Method	94.4	87.3	0.672
	Proposed Method	77.3*†	98.9†	0.845*†
Fibrocalcific	CK Method	81.2	93.0	0.802
	DSP Method	74.8	95.3	0.785
	Proposed Method	97.2*†	91.9	0.816*†
Other	CK Method	93.3	71.0	0.869
	DSP Method	81.4	89.6	0.870
	Proposed Method	93.7†	97.4*†	0.962*†

The proposed method outperformed the previous approaches for all plaques. Using the Wilcoxson signed-rank test, we determined statistically significant differences (p < 0.05) between the methods (*: CK method versus proposed method, †: DSP method versus proposed method). All methods were trained on the entire data set (89 VOIs, 4,819 images) and tested on the held-out set (22 VOIs, 1,737 images).

## Discussion

These first results of hybrid classification/segmentation are promising for clinical treatment planning and research applications. With hybrid features, sensitivities/specificities of fibrolipidic and fibrocalcific plaques over the five folds were 84.8%/97.8% and 91.2%/96.2%, respectively, with substantial F1 score improvement as compared to results before noise cleaning (Table 1). Results from our hybrid method were improved as compared to our previous reports of A-line-based classification using deep learning^27^ and hand crafted features^28^ alone. Kolluru *et al*.^27^ classified A-lines using a deep learning CNN model similar to the one used in this study. Morphological features were not available to the network due to the use of pixel-shifting. In another study, Prabhu *et al*.^28^. analyzed lumen morphology features and for A-line plaque characterization. The current hybrid approach produced very promising results sufficiently quickly (as detailed later) to suggest appropriateness for both research and treatment planning application.

There are important aspects of our hybrid A-line classification/segmentation approach. As described in the Introduction, A-line classification uses the innate, underlying data element in an IVOCT acquisition and incorporates the natural ordering of tissues from the lumen out (e.g., fibrous tissue followed by lipidous tissue). Pre-processing (particularly pixel-shifting) plays a very important role for improved plaque characterization, as reported previously^26^. However, with pixel shifting, the CNN cannot assess lumen border irregularities (as often found in the presence of calcifications) and physics-of-reflection-based attributes. To address this limitation, we extracted hand-crafted, lumen morphology features and combined with the convolutional features. Comparing results between hybrid, lumen morphology only, and deep learning convolutional only classifications (Fig. 8 and Table 2), we found much improved results with hybrid. We found the random forest classifier to be useful in this application. In a preliminary experiment, we compared classification results between decision tree, random forest, and support vector machine methods (not shown). Decision tree had slightly lower metrics compared to the other two. Support vector machine and random forest gave very similar results with the winner depending upon the fold. We chose the latter for a variety of reasons. As compared to support vector machine, random forest was easier to optimize, did not require a separate feature selection step, trained in ∼1/4 the time, and computed at run time in ∼1/3 the time. On our computer system with non-optimized code, our current run-time is only about 1-sec per image (0.05-sec for pre-processing, 0.9-sec for feature extraction, 0.03-sec for classification, and 0.02-sec for post-processing). The proposed method can analyze a high resolution IVOCT pullback in a few minutes. By optimizing coding for speed, the method could be suitable for live-time treatment planning.

Post-processing with CRF remarkably improved classification performance. This is consistent with our previous study using a simpler CNN architecture to classify A-lines^27^. In this case, the overall sensitivity increased by 10–15% after CRF noise cleaning. We observed a similar improvement of traditional metrics (e.g., sensitivity and F1 score) for both fibrolipidic and fibrocalcific plaques. This could be expected as both approaches utilized a single A-line as an input. The improvement with CRF is likely due to the consideration of spatial similarity.

The active learning, relabeling step provided improved classification results, particularly for fibrocalcific plaque where the F1 score was improved from 0.677 to 0.719, a significant difference (p < 0.05). As argued in Results, relabeling was done in a very conservative manner, where the experienced cardiologist had no knowledge of automated or previous manual results. F1 scores between the initial and secondary manual labeling of the two folds (0.965 and 0.937) indicate that overall changes were relatively small. Nevertheless, the relabeling process improved the consistency of labeling, providing the enhanced performance of the automatic scheme for fibrocalcific plaques. This initial experimental result suggests that improvement of noisy labels could lead to additional benefits.

Unfortunately, it is difficult to compare our results to those from previous studies described in the Introduction^7–15,19–21,24–26^ because performance will greatly depend on the case mix and criteria for annotation. Nevertheless, it appears that our method compares favorably to previous studies (Table S1). For example, our method showed higher sensitivity in fibrocalcific plaque than those in other reports on the held-out data set. We have used the largest dataset reported. Our method had the very short processing time (~1-sec per image).

This study has two limitations. First, manually labeled images were used for training. Although we improved some ground truth labels using active learning, this is still an imperfect gold standard. In the future, this limitation could be reduced with extensive active learning or with large data sets using labeling based upon registered cryo-images^42^. Second, we used a relatively simple CNN architecture for convolutional feature extraction. Classification results might be improved with more advanced deep learning models.

In summary, we developed a fully automated, A-line-based plaque characterization method based upon hybrid processing (deep learning and hand-crafted feature machine learning). Hand crafted lumen morphology features provide significant improvement to A-line deep learning, suggesting the importance of these features to determination of IVOCT plaque characterization. The method gives very good plaque classification results and computes in a reasonable time, suggesting that this could be a promising solution for both clinical and research applications.

## Supplementary information

Supplementary file.

## Data Availability

The datasets analyzed during the current study are available from the corresponding author on reasonable request.
