# Supplementary material for: Fully automated plaque characterization in intravascular OCT images using hybrid convolutional and lumen morphology features
Source: Sci Rep. 2020 Feb 13;10:2596. doi: 10.1038/s41598-020-59315-6 (PMC7018759; doi:10.1038/s41598-020-59315-6)
Supplement: Supplementary file 1 — Supplementary file. [file 41598_2020_59315_MOESM1_ESM.pdf]

Fully automated plaque characterization in intravascular OCT images using hybrid convolutional and lumen morphology features

Juhwan Lee[1], David Prabhu[1], Chaitanya Kolluru[1], Yazan Gharaibeh[1], Vladislav N. Zimin[2], Luis A. P. Dallan[2], Hiram G. Bezerra[2], and David L. Wilson[1,3,*]

[1] Department of Biomedical Engineering, Case Western Reserve University, Cleveland, OH, 44106, USA
[2] Cardiovascular Imaging Core Laboratory, Harrington Heart and Vascular Institute, University Hospitals Cleveland Medical Center, Cleveland, OH, 44106, USA
[3] Case Western Reserve University, Department of Radiology, Cleveland, OH, 44106, USA

**dlw@case.edu*

Table S1. Comparison of current method to previous studies. Several entries are missing as some items are not reported. The performance of our current method compares favorably to previous studies. We note that performance greatly depends on case mix and the way that images are annotated.

Learning Methods	Approaches	Lipid (Fibrolipidic)			Calcium (Fibrocalcific)			Processing Time (per frame)	Number of Patients (Frames)
		Sensitivity (%)	Specificity (%)	Accuracy (%)	Sensitivity (%)	Specificity (%)	Accuracy (%)		
Machine Learning	Ughi et al.[7]	-	-	79.5	-	-	72.1	30s	49
	Athanasiou et al.[8]	71.0	-	-	81.0	-	-	40s	22 (556)
	Rico-Jimenez et al.[9]	84.8	90.6	88.2	-	-	-	1.5s	(513)
	Xu et al.[10]	-	-	-	-	-	-	-	29
	van Soest et al.[11]	-	-	-	-	-	-	1-2s	14
	Gargesha et al.[12]	95	93	-	86	86	-	-	-
Deep Learning	Yong et al.[13]	-	-	-	-	-	-	-	28 (5,685)
	Abdolmanafi et al.[14]	-	-	-	-	-	-	-	26 (4,800)
	Abdolmanafi et al.[15]	-	-	-	91.0	98.0	95.0	-	33 (3,149)
	He et al.[19]	25.6	-	-	60.6	-	-	-	22 (269)
	Athanasiou et al.[20]	93.0	98.8	97.6	72.3	98.4	98.1	-	22 (700)
	Gessert et al.[21]	-	-	-	-	-	-	-	49 (4,000)
	Zhang et al.[24]	-	-	-	-	-	-	5s	-
	Abdolmanafi et al.[25]	-	-	-	95.0	84.0	90.0	-	45 (5,040)
	Lee et al.[26]	87.4 (90.1)	89.5 (84.3)	-	85.1 (92.9)	94.2 (76.4)	-	0.3s	55 (4,892)
	Current Method (5-fold cross validation)	(84.8)	(97.8)	-	(91.2)	(96.2)	-	1s	49 (6,556)
	Current Method (Held-out dataset)	(77.3)	(98.9)	-	(97.2)	(91.9)	-		

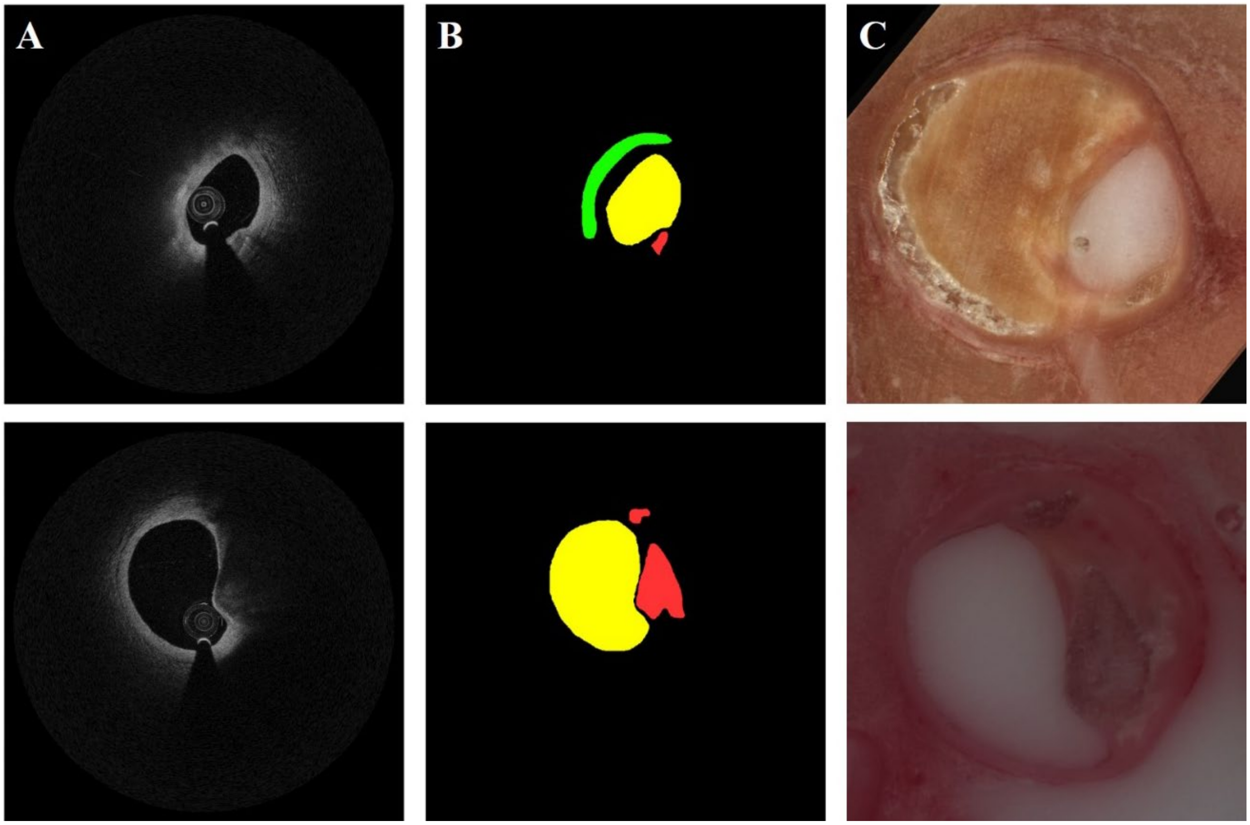

Figure S1. Example annotations and corresponding cryo-images of IVOCT images in *(x,y)* view. Panels show: (A) IVOCT images, (B) corresponding labels, and (C) cryo-images. Colors are yellow (lumen), green (lipidous), and red (calcified).

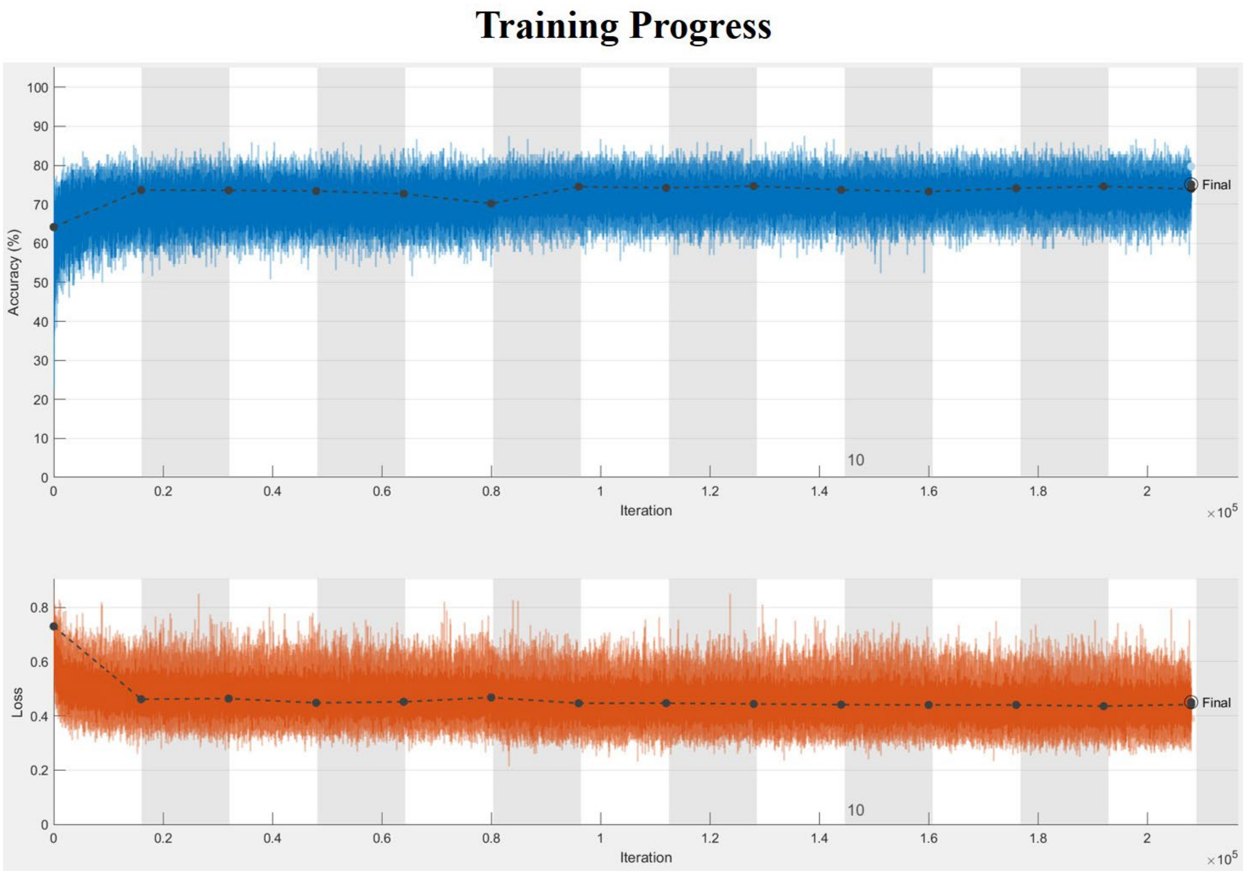

Figure S2. Training progress curve on the one-fold cross validation data set. Exactly the same training options described in the *Implementation and optimization of the CNN and random forest* were used for all folds.

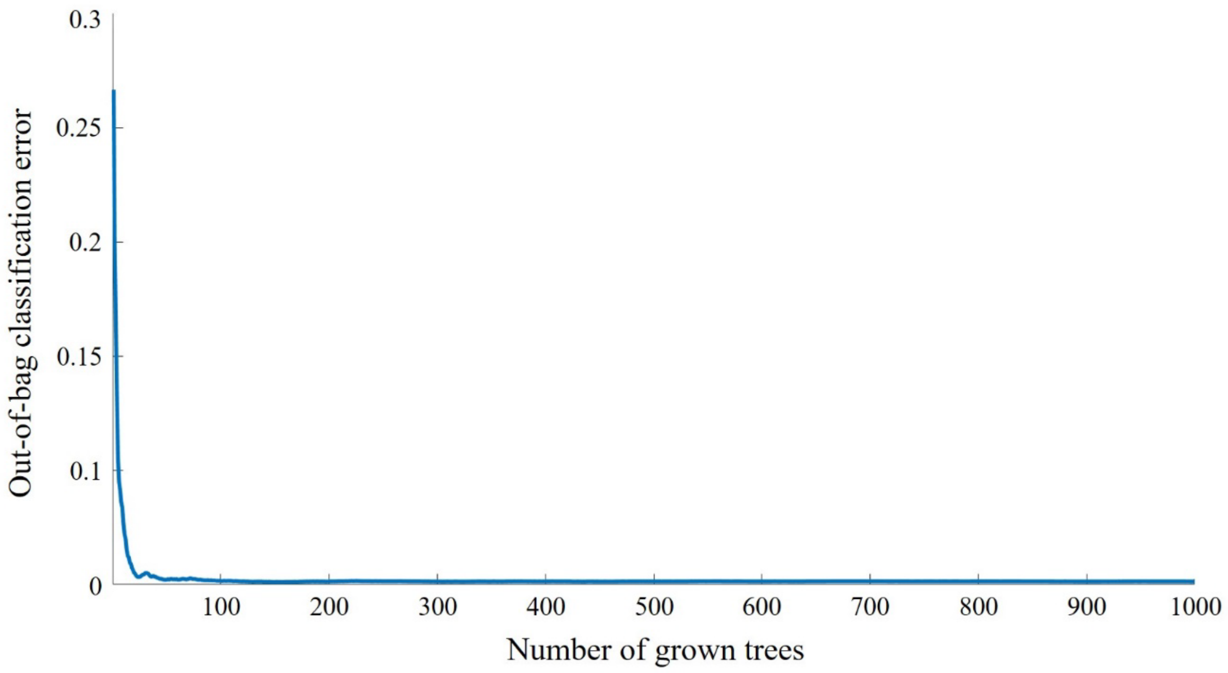

Figure S3. OBB classification errors on the one-fold cross validation data set for varying numbers of grown trees of RF classifier. The values on the x- and y-axis are represented by the number of grown trees and OBB classification error, respectively. OBB errors were kept to a nearly constant value after 250 trees. Therefore, we set the number of trees to 250.

Actual Class \ Predicted Class	Other	Fibrolipidic	Fibrocalcific
Other	547386	7033	30033
Fibrolipidic	3194	105298	27802
Fibrocalcific	3347	582	136877

Figure S4. The confusion matrix of the proposed method obtained on the held-out dataset after noise cleaning.
